# A systematic review on the incidence of influenza viruses in wastewater matrices: Implications for public health

**DOI:** 10.1371/journal.pone.0291900

**Published:** 2024-04-25

**Authors:** Mbasa Dlamini, Luyanda Msolo, Kingsley Ehi Ebomah, Nolonwabo Nontongana, Anthony Ifeanyi Okoh

**Affiliations:** 1 SAMRC Microbial Water Quality Monitoring Centre, University of Fort Hare, Alice, South Africa; 2 Department of Biochemistry and Microbiology, Applied and Environmental Microbiology Research Group (AEMREG), University of Fort Hare, Alice, South Africa; SKUMS: Shahrekord University of Medical Science, ISLAMIC REPUBLIC OF IRAN

## Abstract

Influenza viruses pose a significant public health threat, necessitating comprehensive surveillance strategies to enhance early detection and preventive measures. This systematic review investigates the incidence of influenza viruses in wastewater matrices, aiming to elucidate the potential implications for public health. The study synthesizes existing literature, employing rigorous inclusion criteria to identify relevant studies conducted globally. The essence of the problem lies in the gaps of traditional surveillance methods, which often rely on clinical data and may underestimate the true prevalence of influenza within communities. Wastewater-based epidemiology offers a novel approach to supplementing these conventional methods, providing a broader and more representative assessment of viral circulation. This review systematically examines the methodologies employed in the selected studies, including virus concentration techniques and molecular detection methods, to establish a standardized framework for future research. Our findings reveal a consistent presence of influenza viruses in diverse wastewater matrices across different geographic locations and seasons. Recommendations for future research include the standardization of sampling protocols, improvement of virus concentration methods, and the integration of wastewater surveillance into existing public health frameworks. In conclusion, this systematic review contributes to the understanding of influenza dynamics in wastewater matrices, offering valuable insights for public health practitioners and policymakers. Implementation of wastewater surveillance alongside traditional methods can enhance the resilience of public health systems and better prepare communities for the challenges posed by influenza outbreaks.

## 1. Introduction

Influenza viruses are recognized as the primary causative agents of respiratory tract diseases, resulting in significant health and socioeconomic effects globally [1]. These viruses belongs to the family *Orthomyxoviridae* and are negative sense, enveloped RNA viruses that contain segmented genomes and are categorized into four types, namely influenza A, B, C, and D. All four types of influenza viruses are considered endemic, with types A and B being the most widespread and responsible for causing influenza, commonly known as the flu. Symptoms of the flu typically include chills, fever, headache, sore throat, and muscle pain [2]. In severe instances, influenza can lead to pneumonia, which can be particularly life-threatening for vulnerable populations such as children and the elderly.

In humans, influenza A and B viruses are the most prevalent and impose a significant burden on the global population [3]. Transmission occurs through respiratory droplets released during coughing, talking, and sneezing, as well as through contact with contaminated surfaces followed by touching the nose or eyes [4]. In terms of public health impact, influenza A and B viruses pose the greatest threat, causing seasonal epidemics that affect millions of individuals [5]. While most influenza virus infections are limited to the upper respiratory tract, some cases can be severe and give rise to complications such as pneumonia, acute respiratory distress syndrome, and central nervous system involvement [6]. Numerous studies have drawn attention to the association of influenza A with conditions like encephalitis, febrile seizures, and Guillain-Barre syndrome [1,7–9].

Influenza, a viral pathogen of significant global concern, has long been a subject of intense scientific scrutiny. Its ability to rapidly evolve, evade host immune responses and cause seasonal outbreaks or more severe pandemics underscores the importance of understanding its taxonomy [10]. The paramount significance of influenza taxonomy lies in its role as a roadmap for understanding viral diversity. Influenza A viruses, for instance, exhibit remarkable genetic variability due to frequent point mutations and genetic reassortment. The hemagglutinin (HA) and neuraminidase (NA) glycoproteins, crucial for host cell recognition and viral release, respectively, further subdivide influenza A viruses into distinct subtypes. The constant surveillance and characterization of these subtypes are essential for predicting and preparing for potential pandemics [11].

The treatment and control measures for influenza viruses primarily focus on preventing infection, managing symptoms, and minimizing the spread of the virus. These strategies involve a combination of vaccination, antiviral medications, hygiene practices and public health measures [12]. Vaccination is the most effective preventive measure against influenza. Annual vaccination is recommended, especially for individuals at higher risk of complications, such as young children, elderly individuals, pregnant women, and those with underlying health conditions. Antiviral drugs play a crucial role in supporting the body’s natural defenses against specific viruses that cause diseases. These medications are recognized as essential tools in the prevention and containment of viruses [13]. Currently, there are three recommended antiviral medications, namely oseltamivir, zanamivir, and peramivir, for the treatment of influenza. These drugs act by bolstering the immune system’s ability to combat viral infections and reducing the viral load present in the body [14]. For instance, oseltamivir, an antiviral drug commonly used, specifically targets and treats both influenza A and B viruses. It alleviates flu-related symptoms, resulting in a reduction in severity and a shortened recovery period of one to two days [15]. Moreover, certain studies suggest that early use of antiviral drugs can reduce the risk of mortality among hospitalized individuals with influenza [15].

The influenza virus undergoes various stages throughout its life cycle such as entry into the host cell, entry of viral ribonucleoproteins (vRNPs) into the nucleus, viral genome transcription and replication, export of vRNPs from the nucleus, and assembly and budding at the host cell plasma membrane. This process culminates in the formation of infectious virus particles which are subsequently released into the extracellular environment, allowing them to infect new cells [16].

Wastewater management plays a crucial role in controlling the spread of influenza viruses and other pathogens. There are some key issues related to wastewater management and the circulation of influenza viruses around the world. Firstly, inadequate sanitation infrastructure, for example, many regions, especially in developing countries, lack proper sanitation infrastructure, including sewage treatment plants and wastewater treatment facilities [17]. This can lead to the direct discharge of untreated or partially treated wastewater into rivers, lakes, or coastal areas, potentially contaminating water sources and increasing the risk of spreading influenza viruses. Therefore, wastewater is considered a reservoir of a variety of human pathogens such as influenza viruses that can impact human health and contribute to a range of environmental and health complications [18]. These viruses can enter the water cycle when they are expelled from the fecal matter of infected individuals [19].

This review is premised on the null hypothesis that the incidence of influenza viruses in wastewater matrices is significantly associated with the prevalence of influenza cases in the corresponding populations, and understanding these associations has implications for designing effective public health interventions. This review will be different from the available literature as it will provide a strong emphasis on the practical implications of its findings for public health. This involve recommendations for targeted interventions, policy changes or communication strategies to mitigate the impact of influenza transmission through wastewater.

Certainly! Conducting this systematic review on the incidence of influenza viruses in wastewater matrices and its implications for public health is a valuable and timely research endeavor. There are some potential innovations and contributions that such a review could bring to the literature. Firstly, this systematic review will highlight methodological advancements in the detection and quantification of influenza viruses in wastewater. This include improvements in sampling techniques, laboratory methodologies and data analysis approaches, providing a guide for future studies. Secondly, an innovative aspect could involve correlating the incidence of influenza viruses in wastewater with clinical data on reported cases in the corresponding populations. This could strengthen the link between wastewater surveillance and public health outcomes. Thirdly, the synthesis of data could enable the identification of potential risk factors associated with the presence of influenza viruses in wastewater. This information can be used to develop targeted public health interventions, such as improved sanitation practices or early warning systems. The review may contribute to the development of evidence-based policies related to wastewater management and public health. Lastly, exploring how the information about influenza viruses in wastewater is communicated to the public and policymakers could be an innovative aspect. Effective communication strategies can enhance public awareness and facilitate the adoption of preventive measures.

The global spread of influenza viruses poses significant challenges to public health, necessitating innovative surveillance strategies [20]. Wastewater-based epidemiology has emerged as a promising approach, but the literature lacks a comprehensive synthesis of the incidence of influenza viruses in wastewater matrices and its direct implications for public health. Addressing this gap is crucial for understanding the dynamics of influenza transmission through wastewater and devising effective strategies for early detection and intervention. This systematic review aims to achieve two main objectives. Firstly, it seeks to consolidate existing knowledge on the global incidence of influenza viruses in wastewater matrices, considering variations across geographical regions and diverse environmental conditions. Lastly, the primary objective is to delineate the implications of influenza surveillance in wastewater for public health, informing policies, interventions, and communication strategies. The anticipated outcomes of this systematic review extend beyond the current understanding of influenza surveillance in wastewater. By synthesizing global data, identifying methodological trends, and emphasizing public health implications, the findings will guide future research endeavors. This work will contribute to the development of standardized methodologies, support the integration of wastewater surveillance into public health frameworks, and catalyze interdisciplinary collaborations. Furthermore, the insights garnered from this review may inform the design of targeted interventions, enhancing our ability to mitigate the impact of influenza outbreaks on a global scale.

In summary, this manuscript embarks on a systematic review journey to unravel the intricate relationship between influenza viruses and wastewater matrices, examining global patterns, methodological trends and most importantly, the implications for public health. Therefore, this review offer a comprehensive understanding of the role wastewater plays in influenza transmission dynamics, laying the groundwork for future research, policy development and public health interventions.

### 1.1 Epidemiological evidence of human health risks associated with influenza viruses

The flu is a highly contagious viral illness that can infect people across all age groups. However, certain groups are more vulnerable to its impact, and during pandemics, epidemics, and sporadic outbreaks, it has been associated with significant mortality rates [21]. Pregnant women, children below the age of five (especially those under the age of two), the elderly, and those undergoing chemotherapy and taking steroids are at a heightened risk of developing severe illness or complications if infected [22].

Individuals can become infected with and transmit the Influenza A virus through various means, including direct contact with bodily secretions, inhalation of respiratory droplets, and indirect contact with contaminated objects. This virus is notorious for its significant rates of illness and death, and it is responsible for both seasonal outbreaks and notable historical influenza pandemics [23]. On the other side, human infections with influenza B viruses pose a continuing concern to public health. According to [22] direct contact with an infected individual is the main risk factor for human influenza B infection. For instance, large quantities of viral particles that are harmful to humans are expelled from infected individuals, which are then introduced into wastewater and later cause waterborne infections all over the world [24].

Growing concerns have arisen in light of notable viral outbreaks in recent years, underscoring the potential for a severe viral pandemic originating from influenza viruses present in wastewater matrices. As highlighted by [25], the public’s apprehension is well-founded, considering the catastrophic impact that viral pandemics can have. Notably, historical records indicate that influenza pandemics killed people in absolute numbers than any other disease outbreak in recorded history. Another example, looking at the results of the detection of influenza A and B in the influent of two WWTPs in Germany represents how wastewater matrices are regarded as a reservoir of a variety of influenza viruses and a major cause of global concern [26]. Based on the findings of the study, it was proven that two German cities’ municipal wastewater contained respiratory virus RNA. For example, influenza B virus was detected in 36.0% and 57.7% of the sampled wastewater. It was concluded that more research should be done using more samples from various places to learn more or expand knowledge about the prevalence of influenza in wastewater matrices. Particularly in situations where preventive measures to mitigate the transmission of respiratory viral infections are lacking, it becomes crucial to examine and comprehend the broader implications of influenza viruses present in wastewater.

During the spring of 2022, the City of Ottawa and its neighborhoods experienced an unusual spike in influenza virus activity, which allowed for the collection of wastewater samples containing influenza viruses [27]. The findings of the study were then applied to improve a procedure for measuring influenza virus RNA in wastewater. Their improved method was then used to measure influenza virus RNA in wastewater from the whole city of Ottawa, Ontario, Canada, as well as from three different neighborhoods. Influenza A virus was demonstrated in 10.3% of the samples from treatment plant 1 (WRRF), 3.1% from treatment plant 2, and 0.1% of the samples from plant 3. These results concur with those of another research that identified the influenza A virus in wastewater matrices [28]. Therefore, based on the findings, the processing of wastewater samples and detection of influenza viral signals was performed and it was concluded that wastewater indeed represents a reservoir of numerous pathogens.

**Fig 1:**
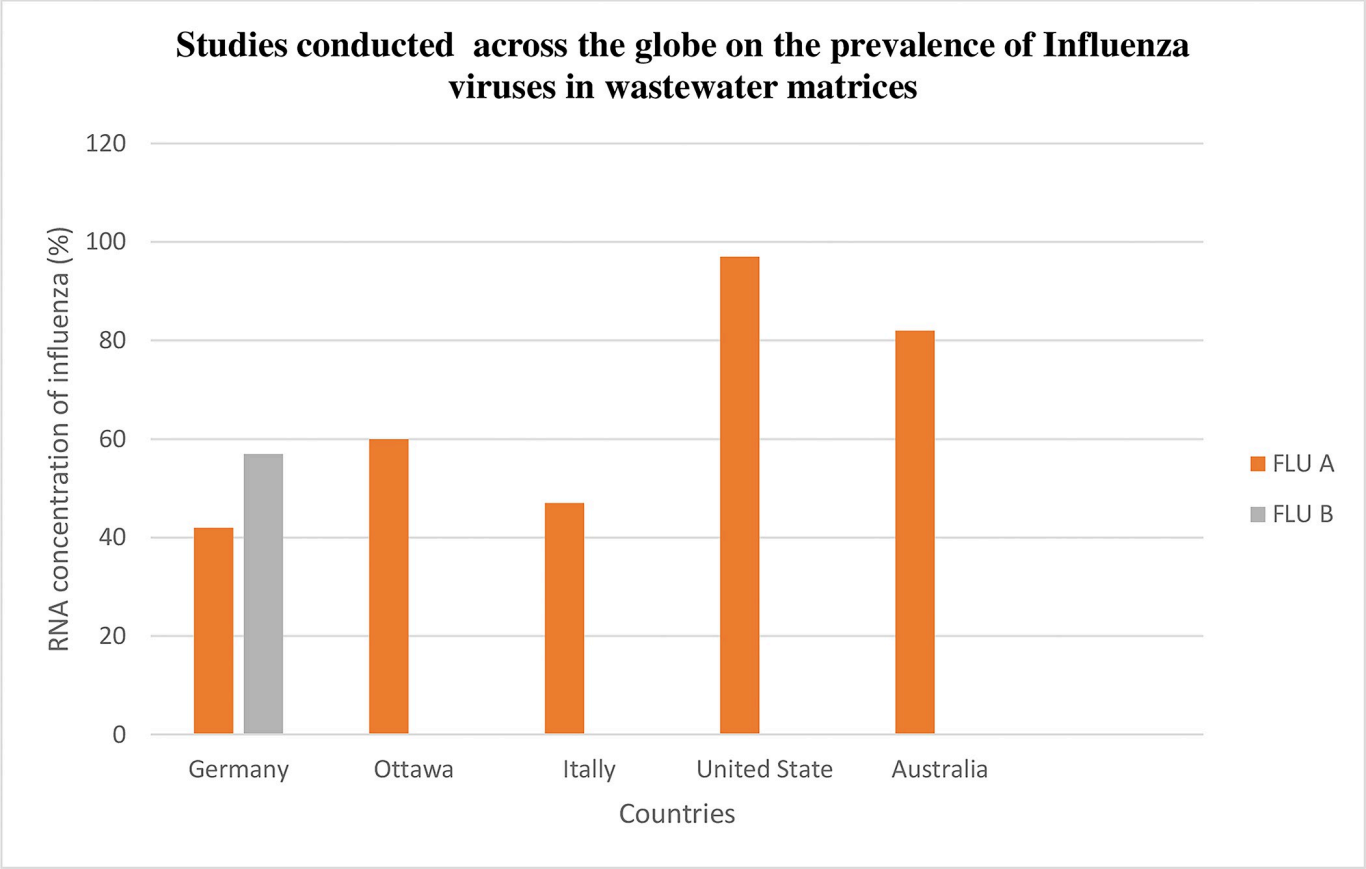
Studies conducted across the globe on the prevalence of influenza viruses associated with wastewater.

To examine the potential of influenza transmission channels, several investigations of the influenza virus in wastewater have been conducted (Fig 1). Few research has described a procedure for successfully detecting influenza virus in wastewaters up to this point [29]. For example, a recent study showed a good correlation between wastewater measurements of influenza A viruses and reported clinical IAV cases seen as part of monitoring systems for athletes at Stanford University and Michigan University’s campus [27]. However, there is a dearth of knowledge on the measurement, trends, and most critically, the relationship between the influenza virus signal in wastewater in cities and neighborhood communities. Therefore, new information linked to influenza WWS is thus necessary to clarify other characteristics of the influenza virus in wastewater matrices. According to [30], wastewater surveillance for influenza possesses several advantageous characteristics, including its ability to provide anonymous, aggregated, cost-effective, and rapid monitoring of a substantial portion of communities. Due to its involuntary contributions, WWS serves as an important tool for public health units or agencies.

### 1.2 How wastewater treatment helps prevent disease

Wastewater treatment is the process of removing, eliminating, or inactivating the majority of contaminants and disease-causing organisms, such as influenza viruses from wastewater [14]. Globally, wastewater poses significant risks of infections, which accounts for around a million fatalities each year, nearly half of which are children and more than 90% of which occur in underdeveloped nations. The main cause of these fatalities is ingesting fecal pathogens from people or animals [22]. Hence, there is a desire among researchers to create and examine criteria based on a clearer framework for determining the danger of influenza viruses in wastewater. A technique known as wastewater-based epidemiology (WBE), which is a tool used to estimate the likelihood of infectious diseases among individuals exposed to materials associated with waste, is becoming more popular as a result of this aim. According to [4], this tool, enables the identification and surveillance of both pandemic and seasonal influenza outbreaks.

### 1.3 Environmental factors influencing the transmission of the influenza viruses

**Fig 2:**
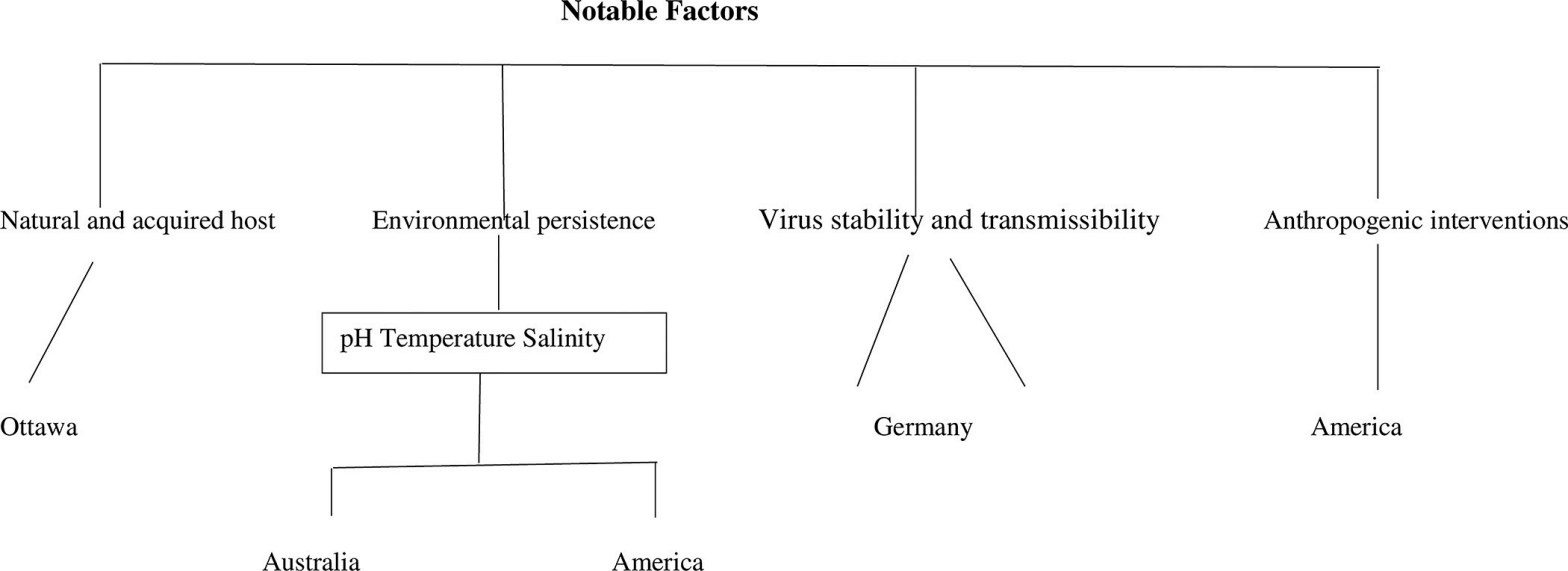
A dendrogram that illustrates some of the notable factors that influence the transmission of influenza viruses in different regions.

Several factors influence the severity and transmission of influenza (Fig 2), including the natural and acquired hosts, viral-host interactions, environmental persistence, virus stability, and anthropogenic interventions [31]. Variations in temperature, humidity, pH, salinity, air pollution, and solar radiation can have an impact on a virus’ ability to survive in varied habitats. In temperate regions with higher latitudes, influenza tends to thrive and spread more effectively in cool and dry conditions, whereas, in tropical and subtropical areas characterized by humid and rainy climates, outbreaks are more prevalent, as observed by [32].

Viruses that target the human respiratory system and give rise to diseases possess diverse mechanisms of transmission. However, they all share the capability to spread among individuals. The transmissibility of these viruses is influenced by the circumstances in which a pathogen and a host come into contact. Various hypotheses have been proposed to elucidate the intricate relationship between temperature, humidity, and the notable seasonality of viruses [33]. These theories encompass alterations in host behavior and adjustments in the virus’s susceptibility to infection and stability under different environmental conditions. High temperatures can cause the denaturation of viral capsid proteins’ secondary structures, while cold temperatures can lead to nucleic acid degradation, for example, disease prevalence tends to decrease as the temperature rises [34].

The transmissibility of viruses can be impacted by ambient humidity, which has implications not only for the stability of the virus but also for the size of respiratory droplets as the water content evaporates. The size of these droplets, in turn, plays a role in determining whether they settle quickly to the ground or remain airborne long enough to be inhaled by a susceptible host [35]. Moreover, the pH levels can influence the transmission of influenza viruses. For instance, when the pH reaches a certain acidic threshold, it triggers a conformational change in the HA protein, exposing the fusion peptide. This critical process initiates the fusion of the virus with the host, leading to the uncoating and the release of the viral genome into the host cell’s cytoplasm [16].

### 1.4 Pathogenesis of influenza viruses

Influenza viruses are known to regularly circulate within human population worldwide, giving rise to seasonal epidemics [36]. As they circulate, these viruses undergo gradual mutations through a process referred to as antigenic drift. Upon respiratory transmission, the virus attaches itself to respiratory epithelial cells in the trachea and bronchi, subsequently penetrating them. This leads to viral replication, destroying the host cell [35].

The pathogenicity of the influenza virus relies on the interplay between viral proteins and host immune responses, encompassing both innate and acquired immunity. This highlights the significance of both viral factors and the host immune system in determining the course of influenza pathogenesis. The immune system serves as a defense mechanism against influenza virus infection. When respiratory epithelial cells or alveolar macrophages are infected by IAV, the single-stranded RNA of the virus is recognized by toll-like receptor (TLR) 7 and retinoic acid-inducible gene-I [37]. Activation of the TLR7 and RIG-I signaling pathways leads to the production of type I interferons and triggers antiviral responses in the host [38].

## 2. Material and methods

The study followed the updated Preferred Reporting Items for Systematic Reviews and Metal Analyses extension for PRISMA guidelines [39]. This review includes the work done from 2012 to 2022 (refer to Table 1).

**Table 1:** Most productive authors in research related to the prevalence of influenza viruses in wastewater milieu from 2012 to 2022 (scopus and google scholar).

AUTHORS	TITLE	SOURCE TITLE	CITED BY	DOCUMENT TYPE	H-INDEX	COUNTRY	PUBLICATION DATE
Bi *et al*., [40]	Simultaneous detection and mutation surveillance of SARS-CoV-2 and multiple respiratory viruses	Medical Sciences	9	Review	12	United State	2021
Boehm *et al*. [41]	Wastewater surveillance of human influenza, metapneumovirus, parainfluenza, respiratory syncytial virus (RSV), rhinovirus, and seasonal coronaviruses during the COVID-19 pandemic	Environmental Science and Health	6	Review	12	USA	2022
Brian *et al*. [42]	A water-focused one-health approach for early detection and prevention of viral outbreaks	One Health	43	Article	20	United State	2019
Brisebois *et al*. [43]	Human viral pathogens are pervasive in wastewater treatment center	Journal of Environmental Science	62	Article	30	China	2018
Dumke *et al*. [26]	Simultaneous Detection of SARS-CoV-2 and Influenza Virus in Wastewater of Two Cities in Southeastern Germany, January to May 2022	International Journal of Environmental Research and Public Health	1	Article	24	Germany	2022
Gaidet *et al*. [44]	Understanding the ecological drivers of avian influenza virus infection in wildfowl: A continental-scale study across Africa	Processing of the Royal Society B: Biological Sciences	109	Article	20	Africa	2012
Ghernaout *et al*. [45]	New insights towards disinfecting viruses	Journal of Water Reuse and Desalination	15	Article	10	Algeria	2020
Kevill *et al*. [46]	Assessment of two types of passive sampler for the efficient recovery of SARS-CoV-2 and other viruses from wastewater	Science of the Total Environment	3	Article	6	United kingdom	2022
Mercier *et al*. [27]	Wastewater surveillance of influenza activity: Early detection, surveillance, and subtyping in city and neighbourhood communities	Scientific Reports The preprint server for Health Science	2	Article	6	Canada	2022
O’Brien *et al*. [47]	A water-focused one-health approach for early detection and prevention of viral outbreaks	One Health	45	Review	20	United State	2019
Ramos *et al*. [48]	A robust, safe and scalable magnetic nanoparticles workflow for RNA extraction of pathogens from clinical and wastewater samples	Global Challenges	10	Article	6	Germany	2021
Ronnqvist *et al*. [49]	Detection Method for Influenza Viruses in Water	International Journal of Environmental Research and Public Health	11	Article	8	Finland	2012
Rusinol *et al*. [50]	Concentration methods for the quantification of coronavirus and other potentially pandemic enveloped virus from wastewater	Current Opinion in Environmental Science and Health	65	Review	6	Spain	2020
Silverman and Boehm [51]	Systematic Review of the Persistence of Enveloped Viruses in Environmental Waters and Wastewater	Environmental Science & Technology Letters	109	Review	12	United State	2021
Sim *et al*. [52]	Future perspectives of wastewater-based epidemiology: Monitoring infectious disease spread and resistance to the community level	Environmental International	431	Review	25	UK	2020
Teirlinck *et al*. [53]	Annual report Surveillance of influenza and other respiratory infections in the Netherlands	Scientific Reports The preprint server for Health Science	15	Article	18	Netherlands	2017
Wigginton *et al*. [54]	Emerging investigators series: the source and fate of pandemic viruses in the urban water cycle	Environmental Science: Water Research & Technology	190	Review	20	United State	2015
Wolfe *et al*. [55]	Wastewater-Based Detection of Two Influenza Outbreaks	Environmental Science and Technology Letters	6	Article	12	United State	2022
Ye *et al*. [56]	Survivability, Partitioning, and Recovery of Enveloped Viruses in Untreated Municipal Wastewater	Environmental Science and Technology	356	Article	30	United States	2016
Zhang *et al*. [57]	Occurrence of various viruses and recent evidence of SARS-CoV-2 in wastewater systems	Journal of Hazardous Materials	35	Review	24	China	2021

### 2.1 Searches and source identification

Searches were performed for the period from March 2012 to December 2022 using google scholar and scopus databases.

### 2.2 Search strategy

Searches were performed using google scholar and scopus databases using the keywords mentioned and a combination of the keywords. Other website such as the World Health Organisation library databases was also used to search for data. The keywords used were based on the terminology normally used in this subject, including: “Wastewater” OR “Epidemiology” AND “Influenza” OR “Viruses”. The searched articles were screened from their titles, abstracts, and full text to select the eligible studies.

### 2.3 Study objective

The objective of this study was to investigate the frequency of detection of influenza viruses in wastewater matrices.

### 2.4 Systematic review

The following inclusion and exclusion criteria were used when selecting the studies to be included in a systematic review.

#### 2.4.1 Inclusion criteria

Studies that measure Influenza viruses in wastewater were included in this study. Articles were limited to those published in English and those conducted in communities or populations, including private/public facilities such as hospitals connected to sewer lines. The studies which provide intelligible information on the method of detecting influenza viruses using: real-time reverse transcriptase quantitative polymerase chain reaction (RT-qPCR) was considered for inclusion in the systematic review. Studies that reported data based on the following metrics were also included: (1) detection of Influenza viruses from wastewater treatment plants (WWTPs); (2) linking data obtained from the WWTPs to RNA prevalence, resulting in an estimated value for community infection.

#### 2.4.2 Exclusion criteria

Summarized publications, abstracts, subscription-based articles without access, newspaper reports and studies using Influenza viruses in other matrices or contexts other than in wastewater, were excluded from this review.

### 2.5 Data extraction

Data was extracted from google scholar and scopus databases by reviewing titles, abstracts for inclusion and exclusion criteria.

### 2.6 Data synthesis

A systematic review and meta-analyses were done. Therefore, as meta-analyses was performed statistical heterogeneity and publication bias were not assessed.

### 2.7 Quality assessment

Included articles were assessed for methodologies bias using the previously described tool by [55]. Items on the tool include the following characteristics: Clarity of sampling approach; consistent use of proper processing for virus concentration; method of virus extraction and detection; presence of viral quantification process, clarity on sampling site(s) and transparency in results (including statistics) presentation.

### 2.8 Justification for database and keyword choices

Google scholar is chosen for its broad coverage while scopus is selected for its comprehensive bibliographic database, advanced search options, and features like citation analysis. Combining these databases can provide a well-rounded and exhaustive search for literature relevant to the research topic. The choice of keywords in a search strategy is crucial for retrieving relevant and comprehensive information.

## 3. Results

### 3.1 Description of studies

**Fig 3:**
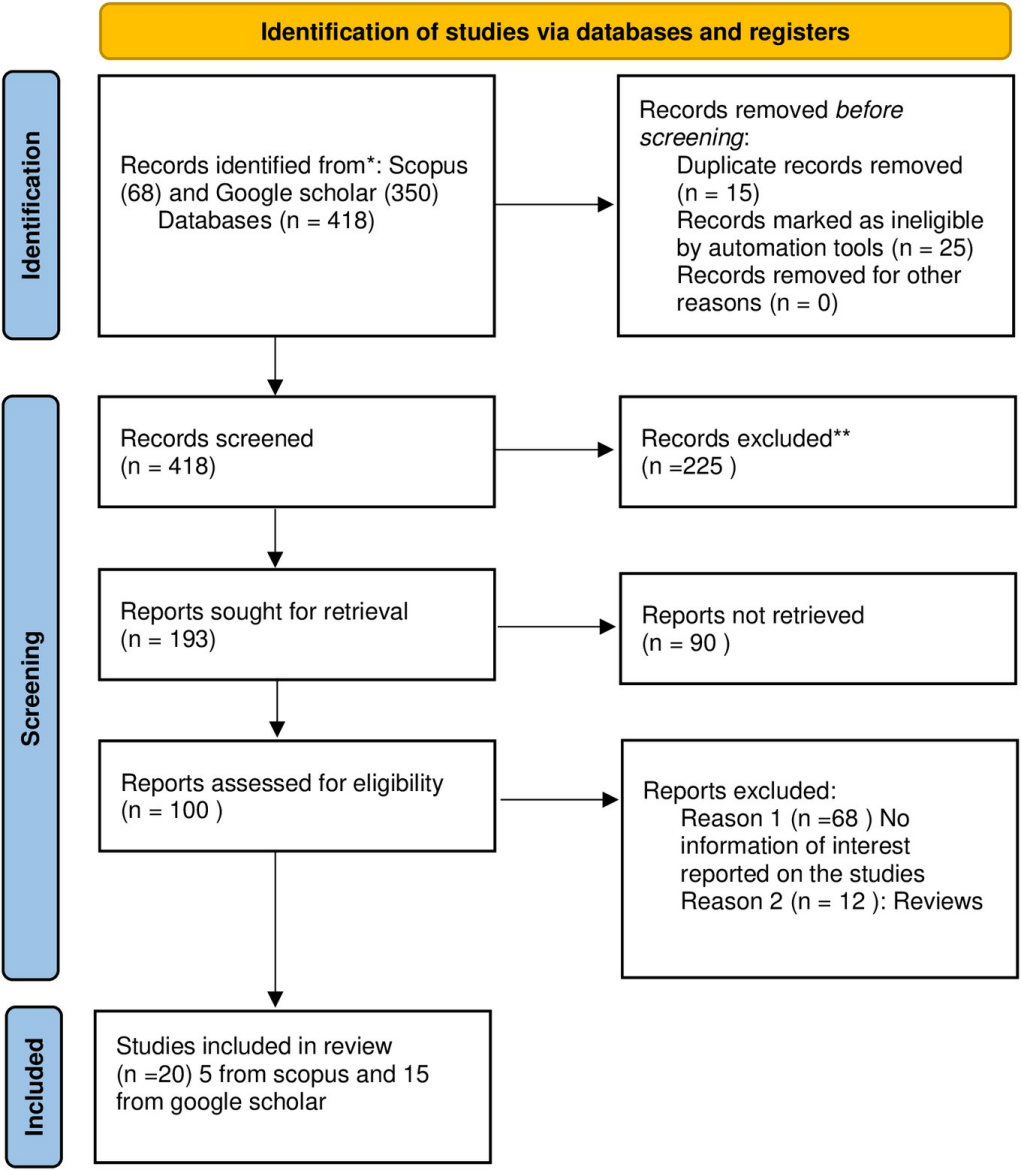
Flow diagram of search, screening, and study selection of systematic review.

Our keyword search found 418 potential articles (68 from scopus and 350 from google scholar). Following further screening, 68 reports were excluded. 20 articles (5 from scopus and 15 from google scholar) were used for data extraction (Fig 3).

### 3.2 Study characteristics

Twenty articles (5 from scopus and 15 from google scholar) were reviewed for qualitative synthesis. Summarized characteristics from each article: authors, country, population size, number of samples collected and PCR methods and targeted genes were used (Table 2). Most studies were conducted in developed countries, whereas only one study was conducted in the African region. Municipal wastewater treatment facilities were primarily used to perform the studies, whereas some studies chose a defined community, such as hospitals, universities, or neighbourhoods.

**Table 2:** Summary of the study details, sampling, and laboratory methodologies.

Author (Year)	Country	Population Size	Number of Samples	Sampling techniques	Sample type (Locations)	PCR methods
Bi *et al*.(2021) [40]	United State	Not mentioned	-	Grab and composite	Raw wastewater (WWTPs)	RT-qPCR
Boehm *et al*.(2022) [41]	United State	1,500,000	-	Grab	Raw wastewater (WWTPs)	RT-qPCR
Brian *et al*.(2019) [42]	United State	290,000	-	Composite	Wastewater (WWTPs)	RT-qPCR
Brisebois *et al*. (2018) [43]	China	Not mentioned	-	Composite	Raw and treated wastewater (WWTPs)	q-PCR
Dumke *et al*. (2022) [26]	Germany	-	273	Composite	Raw wastewater (WWTPs)	RT-qPCR
Gaidet *et al*.(2012) [44]	Africa	-	8413	Composite	WWTPs	RT-PCR
Ghernaout *et al*. (2020) [36]	Algeria	-	-	Grab and composite	Raw and treated wastewater	RT-qPCR
Kevill *et al*. (2022) [46]	United Kingdom	40, 000	201	Passive sampling	Raw wastewater	q-PCR
Mercier *et al*. (2022) [27]	Canada	910, 000	-	24-h Composite	Raw wastewater (WWTPs)	RT-qPCR
O’Brien *et al*. (2019) [47]	United State	290, 000	-	-	-	-
Ramos *et al*. (2021) [48]	Germany	-	-	Composite	Clinical and raw wastewater samples	RT-qPCR
Ronnqvist *et al*.(2012) [49]	Finland	-	-	Grab and composite	Wastewater, lake and river	Real time PCR
Rusinol *et al*. (2020) [50]	Spain	-	-	composite	Raw wastewater	RT-qPCR
Silverman and Boehm (2021) [51]	United State	27	812	Composite	Raw wastewater	RT-qPCR
Sim *et al*. (2020) [52]	United Kingdom	-	-	Grab and composite	Treated and raw wastwater	RT-qPCR
Teirlinck *et al* (2017) [53]	Netherland	500, 000	-	Grab and composite	Treated and raw wastwater	RT-qPCR
Wigginton *et al*.(2015) [54]	United State	398	-	Grab and composite	Raw wastwater	RT- qPCR
Wolfe *et al*. (2022) [55]	United State	130, 000	-	24h composite	Raw wastewater	ddPCR
Ye *et al*.(2016) [56]	United State	115 000	-	Grab	Untreated wastewater	RT- qPCR
Zhang *et al*. (2021) [57]	China	-	-	Grab and composite	Treated and raw wastwater	RT-qPCR

RT-qPCR: Real Time quantitative Polymerase Chain Reaction, WWTPs: Wastewater Treatment Plants, ddPCR: droplet digital Polymerase Chain Reaction.

## 4. Discussion

Twenty studies (5 from scopus and 15 from google scholar) were selected in the current review. Our review provides some insights into the potential risks posed by influenza viruses detected in wastewater matrices which serve as an initial step toward gaining a better understanding of the burden of influenza in the environment. This includes exploring its epidemiology, the consequences of severe influenza infections and by preventing the spread of illnesses caused by influenza viruses through the fortification of water resources.

This study analyzed the existing literature on the incidence of influenza viruses in wastewater matrices, aiming to provide a comprehensive understanding of the potential implications for public health. Our investigation encompassed diverse geographical locations, methodologies, and study periods to ensure a robust synthesis of available evidence. This review revealed a varying prevalence of influenza viruses across different wastewater matrices. The persistence of these viruses in wastewater suggests the potential for continuous exposure and transmission in communities. For example, the City of Ottawa and its neighborhoods experienced an unusual spike in influenza virus activity, which allowed for the collection of wastewater samples containing influenza viruses [27]. The findings of the study were then applied to improve a procedure for measuring influenza virus RNA in wastewater. It was also observed that WWTPs in Germany represents how wastewater matrices are regarded as a reservoir of a variety of influenza viruses and a major cause of global concern [26]. Based on the findings of the study, it was proven that two German cities’ municipal wastewater contained respiratory virus RNA. For example, influenza B virus was detected in 36.0% and 57.7% of the sampled wastewater.

Monitoring influenza viruses in wastewater matrices is a crucial epidemiological and public health model with a number of essential advantages such as (1) Early detection and monitoring- Wastewater surveillance enables the early detection and frequency of influenza viruses in a community, prior to the reporting of clinical cases. This can assist public health authorities track changes over time and can provide valuable insights into the prevalence of the virus in a population [52]. (2) Wastewater monitoring offers a population-level perspective, enabling a more thorough evaluation of the virus’s presence throughout an area or community. This information can help public health authorities to prepare and allocate resources and timeously respond effectively to outbreaks. (3) Early warning system: Monitoring wastewater can act as an early warning system for epidemics that can occur, for example increased public health testing and treatments may be required in the affected region if a significant increase in viral RNA is detected in wastewater [24].

Our systematic review has underscored the significant presence of influenza viruses in wastewater matrices, highlighting the potential of wastewater surveillance as a valuable tool for public health monitoring. Policymakers should consider integrating wastewater surveillance into existing public health strategies to enhance early detection and response capabilities. According to the study done by [41], the detection of influenza viruses in wastewater can serve as an early warning system for potential outbreaks in communities. Implementing policies that enable rapid response and intervention based on wastewater surveillance data can aid in preventing the spread of influenza and minimizing the impact on public health. Our findings suggest variations in the incidence of influenza viruses across different geographical areas. Therefore, policymakers should allocate resources based on the identified high-risk areas, directing interventions and resources where they are most needed. This targeted approach can optimize resource utilization and enhance the effectiveness of public health measures. Effective communication strategies are essential for the successful implementation of public health policies [58]. Policies should include provisions for transparent and timely communication with the public regarding the implications of wastewater surveillance findings. Engaging the community in understanding the importance of these measures can foster cooperation and compliance with recommended interventions.

The scarcity of studies conducted in the African region on the prevalence of influenza viruses in wastewater matrices can be attributed to several factors such as limited infrastructure, data collection challenges, limited research collaboration, low awareness and seasonal nature of influenza [18]. For example, limited infrastructure: Many African countries face challenges in terms of infrastructure and resources for scientific research, including wastewater surveillance. Lack of financial assistance, laboratory facilities and trained personnel can hinder the ability to conduct such studies. Data collection challenges: collecting and analyzing wastewater samples for influenza surveillance requires specialized equipment and expertise. In many African countries, these capabilities may be lacking or underdeveloped [4]. Low awareness: Influenza surveillance in wastewater is a relatively new concept, and there may be a lack of awareness or understanding of its potential benefits among public health officials and researchers in the African region. Seasonal nature of influenza: Influenza infections tends to be seasonal in many regions, including Africa, which can make it more difficult to conduct year-round surveillance and may limit the perceived urgency of such studies [59].

Therefore, to address the scarcity of studies in the African region, it is essential to prioritize capacity building, promote international collaboration, increase awareness of the importance of wastewater surveillance, and allocate resources specifically for this purpose. Investing in research infrastructure and training for local researchers and institutions can help bridge the gap and contribute to a more comprehensive understanding of influenza viral infections in the region. Additionally, raising awareness about the potential benefits of wastewater surveillance for public health can encourage greater research efforts in this area.

## 5. Limitations

The current study had some limitations, the foremost of which was article sample size. The total number of articles we examined was not highly satisfactory in comparison with other scholarly articles published. There were also insufficient studies on public health implications which led to a limited number of studies specifically addressing the public health implications of detecting influenza viruses in wastewater, hindering the ability to draw robust conclusions about the potential risks and necessary public health interventions. We also faced challenges in interpreting (findings) the significance of detected influenza viruses in wastewater in terms of actual health risks, as the presence of the virus does not necessarily equate to active infection or transmission in the community.

## 6. Conclusion

In conclusion, this systematic review has provided a comprehensive analysis of the incidence of influenza viruses in wastewater matrices, shedding light on crucial aspects with significant implications for public health. The synthesis of available literature has allowed for the identification of patterns, trends, and potential areas for further investigation. Our research adds substantial scientific value by consolidating and critically assessing the existing evidence on influenza viruses in wastewater. By employing a systematic approach, we have enhanced the reliability of our findings. The implications for public health are evident, as understanding the prevalence of influenza viruses in wastewater can serve as an early indicator of community outbreaks and aid in the development of targeted public health interventions. One of the notable contributions of our study lies in its focus on specific aspects, methodologies, or populations which distinguishes it from previous reviews. While previous studies have provided valuable insights into the presence of viruses in wastewater, our work specifically addresses (novel aspect), providing a more understanding of the dynamics of influenza viruses in diverse wastewater matrices. Despite the strides made in understanding the presence of influenza viruses in wastewater, our review has identified notable research gaps that further investigation. The lack of standardized methods for virus detection and the limited representation of certain geographical regions are among the gaps identified. These gaps present opportunities for future research to refine methodologies, expand geographical coverage, and provide a more comprehensive understanding of the factors influencing virus prevalence in wastewater matrices. Looking ahead, future research endeavors should aim to standardize methodologies, increase global representation, and explore the potential correlation between wastewater surveillance data and clinical epidemiology. Moreover, advancements in molecular techniques hold promise for more precise virus detection and characterization. By addressing these aspects, researchers can contribute to a more robust evidence base for public health decision-making.

## Supporting information

S1 Checklist PRISMA 2020 checklist. (DOCX)
